# Quantification of Cognitive States via Eye Tracking and Using Artificial Intelligence to Analyze Virtual Reality Learning Experiences

**DOI:** 10.3390/jemr19030050

**Published:** 2026-05-05

**Authors:** Haram Choi, Sanghun Nam

**Affiliations:** 1 Department of Culture and Technology Convergence, Changwon National University, Changwon 51140, Republic of Korea; 20227578@gs.cwnu.ac.kr; 2 School of Meta-Convergence Content, Changwon National University, Changwon 51140, Republic of Korea

**Keywords:** artificial intelligence, cognitive state, eye tracking, virtual reality

## Abstract

Virtual reality (VR) technology provides a high sense of immersion and presence to users and can enhance the engagement and performance of learning. However, the VR learning environment introduces more complex audio–visual stimuli than the traditional multimedia learning environment. These excessive stimuli cause negative effects such as distraction and cognitive overload. To minimize these negative impacts and improve the learning environment, we must evaluate learners’ cognitive states under the VR environment. Cognitive states can be evaluated subjectively (e.g., through questionnaires) or objectively (e.g., using biometric signals). Subjective and objective methods must be used simultaneously, and correlations between them must be analyzed for quantifying objective measures. The accurate detection of cognitive states is challenging for traditional statistical analysis methods, necessitating the exploration of artificial intelligence (AI) techniques that can classify cognitive states. This study develops a VR learning experience evaluation system based on eye-tracking data. Cognitive states during VR learning are classified as cognitive overload, immersion, and distraction. Correlations between each cognitive state and eye-tracking metrics are evaluated, and the possibility of cognitive-state quantification is discussed. An LSTM-based model developed in this study classified cognitive states from eye-tracking data with moderate accuracy (75.60%) under a subject-independent validation setting.

## 1. Introduction

In virtual reality (VR), presence is the subjective feeling of being in a space without physically occupying that space, creating a realistic experience and high immersion. This allows VR to improve the engagement and performance of learners. Accordingly, VR has been used in various learning disciplines, such as engineering, astronomy, biological sciences, medicine, and geology [1,2].

Cognitive Load Theory (CLT) suggests that human working memory has a limited capacity for processing information, and cognitive load may become problematic when the demands of a learning task exceed this capacity. Such states can negatively affect learning performance, highlighting the importance of designing strategies that support effective cognitive processing [3].

Due to its 360° field of view, VR learning environments often present richer and more complex audiovisual information than conventional two-dimensional (2D) multimedia environments. Under certain conditions, this may increase cognitive demands on learners, potentially leading to states such as cognitive overload or distraction, which can interfere with effective learning [4,5,6]. Therefore, from a CLT perspective, it is important to carefully design VR learning environments to manage cognitive demands and support learning performance.

Assessing the cognitive states of learners is essential for avoiding cognitive load problems. Cognitive states are evaluated either subjectively or objectively. Subjective evaluations are self-evaluations, where learners rate their own perceptions of cognitive load. Subjective evaluation methods are generally considered reliable and widely applicable, but they can provide inaccurate information due to individual biases and memory distortions. Moreover, because of their retrospective nature, subjective evaluations cannot be applied to real-time educational settings. Objective evaluations quantify the cognitive loads of learners from measured physiological responses or biometric data. Objective evaluation methods enable real-time analyses of cognitive states and provide quantitative data. However, owing to differences among individual learners, these methods may not consistently reflect cognitive load. Although considered as useful for evaluating cognitive states, they often lack sufficient validity [7]. Therefore, research on cognitive states must combine subjective and objective evaluation methods and examine correlations between them to enhance the reliability of objective measures. By quantifying the level of cognitive load through objective methods, learners’ cognitive load states can be more accurately assessed [8,9,10]. Moreover, traditional statistical methods may not fully capture individual variability or complex patterns in biometric data. In this regard, artificial intelligence (AI) techniques may provide additional capabilities for identifying patterns related to cognitive states from multimodal signals [11].

This study introduces an eye-tracking-based system for evaluating VR learning experiences. The system assesses the learners’ cognitive states and identifies potential cognitive load problems in VR learning environments. The eye-tracking data corresponding to different cognitive states—cognitive overload, immersion, and distraction—collected by the developed system were evaluated in conjunction with cognitive-state evaluation scales. The eye-tracking data were correlated with the evaluation scale data to determine the possibility of quantifying cognitive states. In addition, an AI model was developed to classify and quantify cognitive states based on eye-tracking data in the VR learning environment.

## 2. Related Work

Grounded in the cognitive structure of learners, Cognitive Load Theory (CLT) is based on psychological research and focuses on improving the processing and understanding of learning materials. CLT aims to analyze the sources of cognitive load and to develop instructional strategies that support effective learning. In CLT, cognitive load is typically categorized into three types: intrinsic cognitive load (IL), extraneous cognitive load (EL), and germane cognitive load (GL). IL is associated with the inherent complexity of learning materials, EL refers to unnecessary cognitive load caused by suboptimal instructional design, and GL is related to cognitive processes involved in schema construction and meaningful learning. Considering the limitations of human working memory, instructional strategies should aim to manage IL, reduce EL, and promote GL [3].

In immersive environments such as virtual reality (VR), learners are exposed to rich and often complex multisensory stimuli. These environments may give rise to diverse cognitive states, such as cognitive overload, immersion, and distraction, depending on task demands and environmental factors. While these states are not direct equivalents of IL, EL, and GL, they can be interpreted in relation to different aspects of cognitive load. For example, cognitive overload may emerge when task complexity exceeds the learner’s processing capacity, distraction may arise from irrelevant stimuli that interfere with attention, and immersion may reflect efficient engagement with task-relevant information. Therefore, rather than directly mapping these cognitive states onto the components of CLT, this study adopts CLT as a theoretical lens to interpret how different VR cognitive states may influence learners’ cognitive processing.

Unlike traditional 2D-based multimedia learning environments, VR provides a 3D-expanded space with complex audiovisual stimuli. While such environments offer enhanced immersion, they may also introduce additional cognitive demands. In some cases, these demands may exceed the learner’s processing capacity, leading to cognitive overload, or may divert attention through irrelevant stimuli, resulting in distraction. From the perspective of Cognitive Load Theory (CLT), these effects can be interpreted as influencing different aspects of cognitive load. For instance, complex or poorly structured learning materials may increase the inherent processing demands placed on learners, while irrelevant sensory stimuli may interfere with efficient allocation of attention. Therefore, rather than directly mapping specific VR cognitive states onto the components of CLT, this study adopts CLT as a theoretical lens to understand how instructional design in VR environments may influence learners’ cognitive processing. Accordingly, VR learning environments should be carefully designed to manage cognitive demands and support effective learning outcomes [6].

To address cognitive problems in VR, methods for analyzing and evaluating cognitive states must be developed. Common self-evaluation tools used for subjective evaluations are cognitive load rating scales, interviews, etc. For example, NASA Task Load Index (NASA–TLX) developed by NASA is a validated instrument for measuring subjective cognitive load and has been widely used in numerous cognitive load assessment studies [12]. Subjective evaluation methods are generally easy to apply across studies. Moreover, because these methods rely on post-task reporting, they cannot be used to assess cognitive load in real time during the learning process. Biometric signals can be used for objective evaluations, and among them, eye movement data obtained through eye-tracking technology have been widely used in cognitive load assessments. This is because eye movements effectively reflect changes in cognitive states in response to visual stimuli, providing quantitative measurements of the cognitive load level and real-time evaluations of cognitive states. Eye-tracking technology is a noninvasive method and is suitable for the VR environment because eye-trackers can be embedded or attached to VR hardware [13]. Although objective evaluation methods monitor cognitive states in real time and provide reliable, quantifiable data, they cannot consistently represent an individual’s subjective cognitive load state. Therefore, they cannot sufficiently quantify cognitive states. Research must combine subjective and objective evaluation methods and explore correlations between them. This will help enhance the reliability of objective evaluation methods for quantifying cognitive load states, allowing the real-time assessments of learners’ cognitive load states in the VR learning environment [7,8,9,10]. Using eye-tracking technology, Latini et al. analyzed the impact of indoor and outdoor greenery elements such as plants on the visual attention, distraction, cognitive load, and performance of learners in the VR environment. They measured eye-tracking data, such as the number of blinks, pupillary dilation, fixation count, fixation duration, and saccade count under three visual conditions of indoor and outdoor greenery elements. Additionally, they found that eye-tracking data were correlated with cognitive load via a cognitive test. Specifically, cognitive load was positively correlated with the fixation count, fixation duration, and saccade count but was not significantly correlated with the number of blinks or pupillary dilation [14]. Using the fixation count and fixation duration as the key indicators, Liu et al. analyzed whether fixation-related eye-tracking metrics could distinguish and evaluate perceptual and cognitive loads. They revealed that in the environment with a high perceptual load, the fixation duration decreased whereas the fixation count increased. In contrast, in the environment with a high cognitive load, the fixation duration increased whereas the fixation count decreased. That is, perceptual load and cognitive load exerted opposite effects on the fixation indicators, demonstrating that fixation metrics could distinguish and evaluate both load types [13]. Negi and Mitra analyzed whether the fixation duration alone could sufficiently analyze the learning process. They also proposed a triangular model of the fixation duration to examine whether the fixation duration varied with the cognitive states of learners. They found that average fixation durations of <150, 300–500, and >1000 ms indicated a distracted state, a focused state, and a confused state, respectively [15].

Statistical methods often yield large individual differences when quantifying the cognitive load from biometric signals and can be independently influenced by variations in situational factors. The difficulty of observing consistent tendencies in cognitive states increases the difficulty of accurate detection. Meanwhile, AI techniques can identify patterns in eye-tracking data corresponding to different cognitive states, enabling effective classification and prediction of those states [11,16]. Daniel and Kamioka proposed a system that detected the focus states of learners in a multimedia distance learning environment. Using machine learning techniques, their system analyzed four eye-tracking data—fixation count, fixation rate, fixation duration, and saccade length—to detect the concentration state. If the eye-tracking data exceed predefined thresholds, indicating that the learner is not focused on the learning content, the system notifies the instructor of the learner’s attention state. The authors found that multilayer perceptron achieved higher classification accuracy than the sequential minimum optimization and J48 decision tree classifiers. Moreover, among four eye-tracking features deemed appropriate for detecting learners’ attention states, fixation duration was identified as the most important classification feature [11]. The machine learning system of Asish et al. used eye-tracking technology to detect distracted students in the VR learning environment. Learners were then classified into different levels of distraction (low, medium, or high). Among several classification models—convolutional neural network (CNN), long short-term memory (LSTM), CNN–LSTM, random forest, extreme gradient boosting (XGBoost), k-nearest neighbor, and linear discriminant analysis—random forest most accurately classified distraction levels [16]. Gao et al. analyzed the gender-based differences in computational thinking abilities utilizing eye-tracking technology and AI in the VR learning environment. They also trialed several machine learning models: support vector machine, logistic regression, random forest, XGBoost, and light gradient boosting machine (LightGBM). Among these models, LightGBM maximized the classification accuracy [17].

Although the correlations between eye-tracking metrics and cognitive load have been well researched and AI has been applied to the classification and prediction of cognitive states, few studies have analyzed the detailed types of cognitive states within VR learning environments. As different cognitive problems such as distraction and cognitive overload may arise from various cognitive load sources, these states must be separately distinguished and evaluated. To this end, the present study analyzes the patterns of eye-tracking metrics under different types of cognitive load in the VR learning environment and examines the correlation between eye-tracking data and cognitive-state evaluation scales. In addition, it seeks to classify and quantify the possible cognitive states in the VR learning environment using AI techniques.

## 3. Research Methods

When designing multimedia learning materials, such as those used in VR, unfamiliar and complex learning contents can increase IL, thereby intensifying cognitive overload. Meanwhile, audio–visual stimuli irrelevant to instructional contents can increase EL, increasing distraction [16,18]. The cognitive states of learners should be analyzed in VR learning environments to reduce learners’ cognitive overload and distraction while increasing their GL. Such environments enhance the learning performance, concentration, and immersion of learners. Methods for analyzing cognitive states can be divided into subjective and objective evaluation methods. A proper investigation must employ both classes of methods in parallel, identify the correlation between them, and quantify cognitive states through objective evaluation methods [8,9,10]. In this study, cognitive states during VR learning were classified into cognitive overload, immersion, and distraction. Each cognitive state was evaluated through subjective and objective evaluation methods. The correlation between subjective and objective methods for each cognitive state was analyzed to enhance the objectivity of using objective measures and examine the potential of quantifying cognitive states during VR learning.

Eye-tracking data were selected as the objective evaluation data for assessing cognitive states. Eye-tracking indicators related to cognitive load were categorized into two metrics used in previous studies of cognitive load: fixation and saccade. Fixation refers to the duration of gaze on a specific area when processing visual information. Metrics such as fixation duration and fixation count are indicators of the degree of cognitive load, level of comprehension, and level of attention. Saccade refers to rapid eye movement between fixations. Metrics such as saccade duration and saccade count are indicators of the visual search behavior, information processing, and cognitive activity [7,19]. The eye-tracking data are summarized in Table 1.

The subjective evaluation tools used to assess cognitive states were cognitive-state evaluation scales. To evaluate cognitive overload, we adopted the NASA–TLX evaluation scale, which evaluates workload along several dimensions. Created for mental and physical load assessments in aircraft pilots, the NASA–TLX scale is also used in virtual and augmented reality fields [20,21]. NASA–TLX consists of six subscales (Mental Demand, Physical Demand, Temporal Demand, Performance, Effort, and Frustration), each evaluated on a 21-point scale with higher scores indicating a higher perceived workload (Table 2).

Immersion was assessed using the Flow State Scale (FSS). In this study, immersion is operationalized as a relatively neutral and balanced engagement state, characterized by sustained attention with minimal distraction and manageable cognitive demand, rather than a fully developed high-intensity flow state. The FSS has been widely applied across domains such as sports, arts, web browsing, internet usage, and VR. The version adopted in this study is derived from Jackson, Eklund, and Martin’s Short FSS (S-FSS-2) and Core FSS (C-FSS-2), which have been previously applied to VR-based learning contexts [22,23]. The questionnaire consists of 10 items, each evaluated on a five-point Likert scale (Table 3). In this context, the FSS was used to assess relative levels of focused engagement across experimental conditions, rather than to confirm the presence of a full flow experience. A higher total score indicates a higher level of engagement under these operational conditions.

Distraction was evaluated on the Distractibility subscale of the Attention Questionnaire, which also assesses Concentration Ability and Arousal. The Distractibility subscale consists of 13 items assessing distraction or diversion from attention (Table 4). Each item is evaluated on a seven-point Likert scale with higher scores indicating a higher level of distraction [24].

The overall cognitive state and different types of cognitive load were evaluated on the Multidimensional Cognitive Load Scale for Virtual Environments (MCLSVE), a validated scale that evaluates the three dimensions of cognitive load in virtual environments. The MCLSVE consists of three subscales, IL, EL, and GL, each evaluated on an 11-point Likert scale with higher scores indicating higher levels of the corresponding cognitive load state (Table 5). The IL subscale, which is related to cognitive overload, evaluates the complexities of the learning concepts and definitions along with the overall complexity. The EL subscale, which is related to distraction, evaluates the clarity of the materials used for learning, the complexity of the virtual environment, and the interaction methods. The GL subscale, which is related to immersion, assesses the learner’s improvement in understanding the learning content [25].

## 4. An Eye-Tracking System for Evaluating VR Learning Experiences

**Figure 1:**
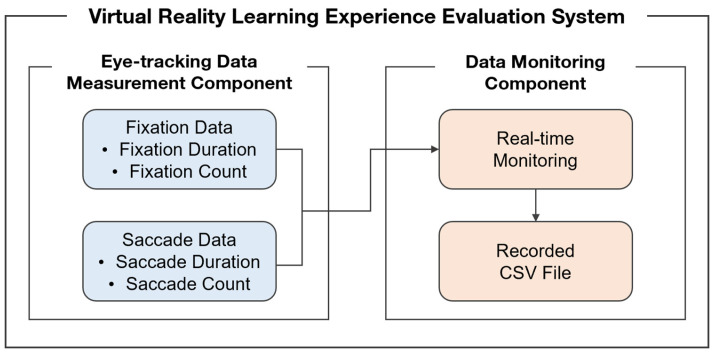
Structure of the system developed for evaluating VR learning experience.

To objectively evaluate the cognitive states of learners using eye-tracking technology, a VR learning experience assessment system was developed. As shown in Figure 1, the system comprised an eye-tracking data measurement component and a data monitoring component. The eye-tracking hardware was the Vive Pro Eye by HTC with an eye-tracking accuracy of 0.5–1.1° within a 20° field of view, an eye-tracking data output frequency of 120 Hz, and a 110° trackable field of view [26]. The VR learning experience evaluation system was developed using Unity (2022.3.10f1), and the eye-tracking data measurement component was developed using the Tobii XR SDK.

**Figure 2:**
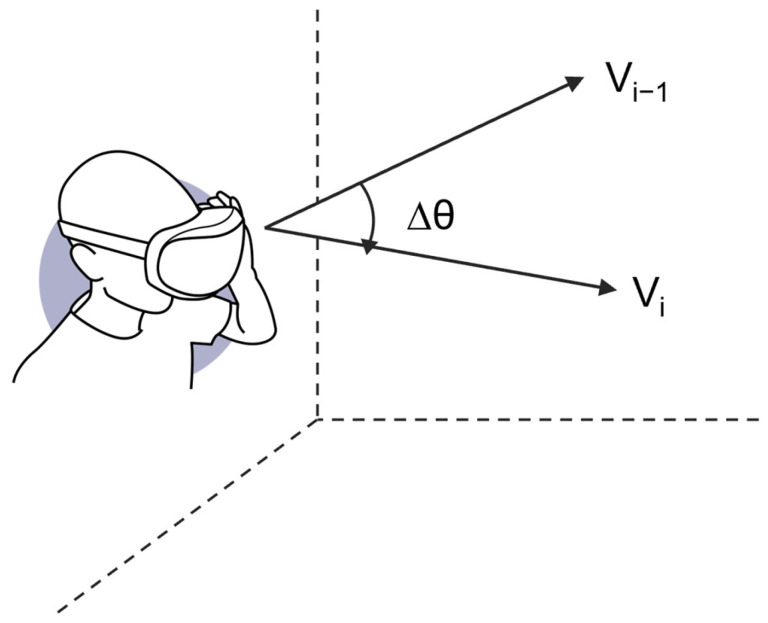
Angular difference between two gaze direction vectors (Δ*θ*).

The eye-tracking data measurement component was developed based on the velocity-threshold identification (I–VT) algorithm to detect fixations and saccades, which are used for evaluating cognitive states. The I–VT algorithm identifies eye movements by calculating velocity v between gaze points in each gaze data sample. If the calculated velocity is below the predefined threshold, that sample is classified as a fixation; otherwise, it is classified as a saccade. Eye movement velocity in the VR environment is calculated from the angular difference Δθ between consecutive gaze direction vectors and the time interval of the eye moment [27,28]. As shown in Figure 2, the eye-tracking data measurement component receives the normalized vector for gaze direction from the API of Tobii XR SDK to calculate the angular difference (Δ*θ*) between the previous gaze direction vector (V_i−1_) and current gaze direction vector (V_i_).

The time Δ*t* between two gaze direction vectors is calculated by subtracting the timestamp *t*_i−1_ of the previous frame from that *t*_i_ of the present frame, where *t*_i_ denotes the current time. Gaze velocity is then calculated as

(1) $$v=\frac{\Delta \theta }{\Delta t}$$

As shown in Table 6, velocity and duration thresholds were defined to accurately detect fixations and saccades from eye-tracking data. These thresholds were selected based on prior studies that specifically addressed eye-tracking in VR environments [17,29], where head-mounted displays (HMDs) introduce additional variability due to head movements. In VR settings, head motion can influence gaze measurements by introducing noise into eye-tracking signals. Therefore, the selected thresholds were chosen to minimize such effects by adopting empirically validated criteria that account for head movement in immersive environments.

In addition to velocity thresholds, duration thresholds were applied to improve the reliability of gaze classification, as velocity alone may lead to misclassification. The duration criteria were based on established eye-tracking literature [30]. Accordingly, fixations were identified when gaze velocity was below 30°/s and the duration ranged between 100 ms and 500 ms. Saccades were detected when gaze velocity exceeded 60°/s, with durations between 30 ms and 80 ms.

**Figure 3:**
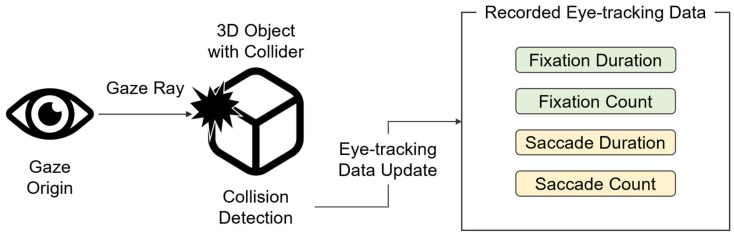
Diagram of the eye-tracking data collection method.

The eye-tracking data were collected using the raycast method of Unity (Figure 3). To track whether a learner gazes at a specific object, the type and size of the collider were configured for the object, which was set as the area of interest (AOI). A collision was detected when the gaze ray originating from the learner’s eye collided with the AOI. At this time, the system updated the eye-tracking data (fixation duration, fixation count, saccade duration, and saccade count) of the collided object. The duration was recorded as the interval between the start and end of the collision, and the count was recorded once each at the start and end of collision.

**Figure 4:**
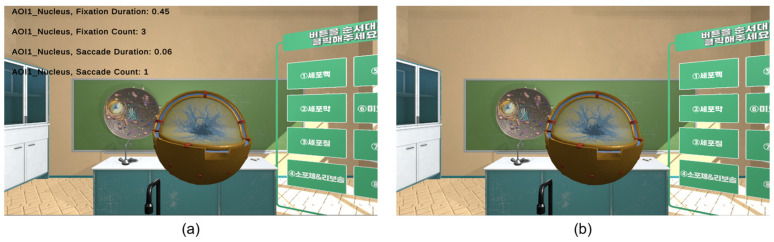
Eye-tracking data monitoring screens: (**a**) the researcher; (**b**) the learner.

Composed of real-time monitoring and data recording functions, the data monitoring component enables researchers to observe learners’ eye-tracking data in real time, thereby facilitating analysis of their cognitive states. The real-time monitoring function was designed for the eye-tracking data at the AOI of the learner. The researcher’s screen displayed the type of AOI, fixation duration, fixation count, saccade duration, and saccade count via a text user interface (Figure 4a), which was invisible on the learner’s screen (Figure 4b). The learner’s cognitive state after learning was analyzed using the data recording function. When the program ends, the eye-tracking data collected in real time were recorded as time-series data and saved in the CSV format.

## 5. Experiment for Quantifying VR Cognitive States from Eye-Tracking Data

This section experimentally analyzes cognitive states induced in the VR learning environment and the potential for quantifying these states using the eye-tracking data. The cognitive overload, immersion, and distraction states in VR were evaluated in separate experimental environments. In this experiment, cognitive-state evaluation scales were used to verify whether the intended cognitive states were induced in each environment, and the tendency of the eye-tracking data was analyzed for each state. In addition, the possibility of quantifying cognitive states was determined by correlating the eye-tracking data with the cognitive-state data.

VR technology is utilized in various educational fields and is particularly suited for applications in biological sciences owing to the abstract and difficult-to-visualize concepts [31]. Therefore, biological science was selected as the VR educational subject in the present experiment. The biological science content was designed to help learners understand the functions and organelles of animal cells. The educational material was based on the official curriculum of the Ministry of Education of the Republic of Korea, referencing elementary and middle-school textbooks depending on the level of learning difficulty [32,33]. AOIs were the organelles of animal cells, namely, the nucleus, cell membrane, cytoplasm, endoplasmic reticulum, ribosome, Golgi apparatus, mitochondria, lysosome, and centrosome.

Cognitive overload, immersion, and distraction were analyzed in different virtual environments, each designed with specific elements that induced the intended cognitive state (Table 7).

**Figure 5:**
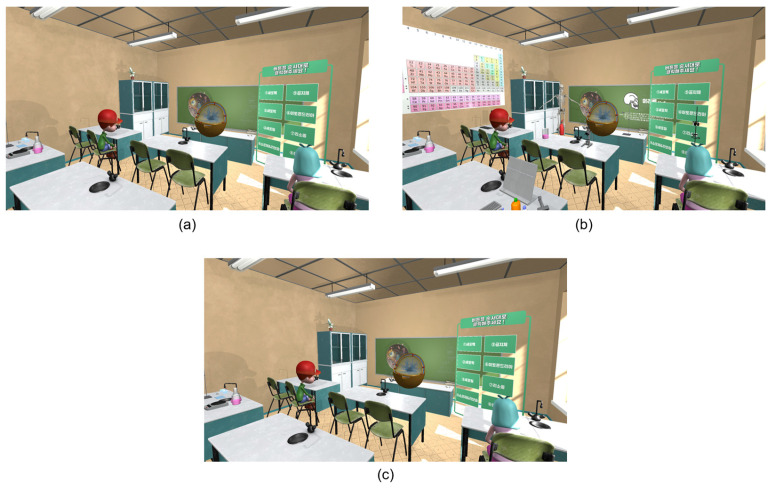
Experimental virtual environments designed to induce different cognitive states: (**a**) cognitive overload; (**b**) distraction; (**c**) immersion.

Cognitive overload can occur when a learner is presented with excessive information or highly complex learning material [34,35]. Accordingly, the educational content in the cognitive overload environment was designed to be more complex and demanding than in other environments, while excluding audio–visual distractions to isolate cognitive load effects (Figure 5a). In the distraction environment, learners were exposed to audio–visual stimuli unrelated to the main learning content [16,33], with the intention of diverting their attention. Visual distractors included irrelevant objects (e.g., periodic table, human skeleton model), and auditory distractors included background noise such as student chatter, smartphone notifications, ringtones, and traffic sounds (Figure 5b). To prevent cognitive overload in this condition, the difficulty level of the educational content was reduced. In contrast, the immersion state was designed to represent a relatively balanced and stable engagement state, positioned between cognitive overload and distraction. To achieve this, both distractive elements and excessive task difficulty were minimized, and concise, easy-to-follow educational content was presented (Figure 5c). This design was intended to support sustained attention without inducing either cognitive overload or external distraction.

**Figure 6:**
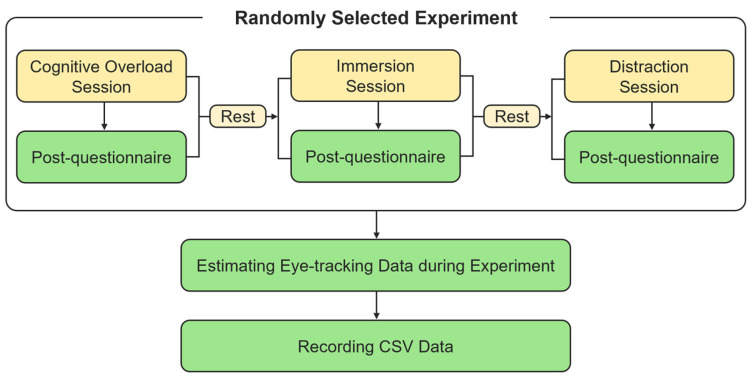
Flowchart of the experimental procedure.

This study was approved by the university’s Institutional Review Board (IRB) (Approval No. 7001066-202412-HR-083). Twenty-six participants were recruited through university communities and social media platforms. All participants had normal or corrected-to-normal vision and experienced no visual discomfort. Participants wore a Vive Pro Eye head-mounted display, which includes a built-in eye tracker. The eye-tracking data were measured at a gaze data output frequency of 120 Hz. All individual participants were presented with the approved experimental protocol and participant consent form from the IRB, and all participants provided informed consent to take part in the study. Figure 6 is a flowchart of the experimental procedure. First, the demographic and vision information of the participants were collected through a prequestionnaire. All consenting participants were briefed on the experiment. Following eye calibration, each participant performed the experiment in three sessions: cognitive overload, immersion, and distraction. To minimize the influence of head movement on eye-tracking measurements, participants were instructed to keep their head movements as stable as possible throughout the experiment. In addition, to reduce potential order effects, the sequence of the experimental sessions was randomized for each participant. After each experimental session, the participants completed a post-questionnaire, followed by a break at regular intervals before proceeding to the next session. The cognitive states of the participants were assessed through a post-questionnaire after each session, which included cognitive overload, immersion, and distraction evaluation scales. The participants’ eye-tracking data were measured in real time during the experiment and saved in CSV format at the end of each session.

The NASA–TLX, FSS, Distractibility, and MCLSVE were assessed across the three experimental sessions. As the same participants completed all states, a repeated-measures analysis of variance (RM-ANOVA) was conducted to examine differences in cognitive-state scores across the three experimental environments. This analysis allowed for the assessment of whether each environment successfully induced distinct cognitive states while accounting for within-subject variability.

Descriptive statistics for NASA–TLX scores across states are presented in Table 8. The cognitive overload state showed the highest mean score (M = 8.97, SD = 5.39), followed by the distraction state (M = 6.02, SD = 2.55) and the immersion state (M = 5.26, SD = 2.29). As shown in Table 9, the RM-ANOVA revealed a significant effect of state on NASA–TLX scores, F (2, 50) = 7.95, *p* = 0.001. As the assumption of sphericity was violated, Greenhouse–Geisser corrections were applied, which remained significant, F (1.42, 35.50) = 7.95, *p* = 0.004, ηp^2^ = 0.241. Bonferroni-corrected post hoc comparisons (Table 10) indicated that the cognitive overload state resulted in significantly higher NASA–TLX scores than both the immersion state (*p* = 0.009, d = 0.897) and the distraction state (*p* = 0.046, d = 0.700). No significant difference was observed between the immersion and distraction states (*p* = 0.621).

Descriptive statistics for FSS scores across states are presented in Table 11. The immersion state showed the highest mean score (M = 4.269, SD = 0.633), followed by the cognitive overload state (M = 3.804, SD = 0.541) and the distraction state (M = 2.992, SD = 1.007). As shown in Table 12, the RM-ANOVA revealed a significant effect of state on FSS scores, F (2, 50) = 23.42, *p* < 0.001. As the assumption of sphericity was violated, Greenhouse–Geisser corrections were applied, which remained significant, F (1.34, 33.49) = 23.42, *p* < 0.001, ηp^2^ = 0.484. Bonferroni-corrected post hoc comparisons (Table 13) indicated that all pairwise differences between states were statistically significant. Specifically, the immersion state. yielded significantly higher FSS scores than both the cognitive overload state (*p* < 0.001, d = 0.79) and the distraction state (*p* < 0.001, d = 1.51), and the cognitive overload state also showed significantly higher scores than the distraction state (*p* = 0.003, d = 1.00).

Descriptive statistics for distractibility scores across states are presented in Table 14. The distraction state showed the highest mean score (M = 5.45, SD = 1.16), followed by the cognitive overload state (M = 2.66, SD = 0.94) and the immersion state (M = 2.25, SD = 0.93). As shown in Table 15, the RM-ANOVA revealed a significant effect of state on distractibility scores, F (2, 50) = 99.74, *p* < 0.001. As the assumption of sphericity was violated, Greenhouse–Geisser corrections were applied, which remained significant, F (1.43, 35.83) = 99.74, *p* < 0.001, ηp^2^ = 0.800. Bonferroni-corrected post hoc comparisons (Table 16) indicated that the distraction state yielded significantly higher distractibility scores than both the immersion state (*p* < 0.001, d = 3.04) and the cognitive overload state (*p* < 0.001, d = 2.64). In addition, a significant difference was observed between the immersion and cognitive overload states (*p* = 0.047, d = 0.44), although the effect size was relatively small.

Descriptive statistics for the MCLSVE subscales (intrinsic load, extraneous load, and germane load) across states are presented in Table 17. The cognitive overload state showed the highest intrinsic load (M = 5.76, SD = 2.46), followed by the distraction (M = 4.45, SD = 2.16) and immersion states (M = 3.51, SD = 2.30). In contrast, extraneous load was highest in the distraction state (M = 2.80, SD = 1.89), followed by the cognitive overload (M = 1.82, SD = 1.47) and immersion states (M = 1.34, SD = 1.67). Germane load was highest in the immersion state (M = 7.66, SD = 2.23), followed by the cognitive overload (M = 6.82, SD = 1.96) and distraction states (M = 5.17, SD = 2.65).

As shown in Table 18, the RM-ANOVA revealed significant effects of state on all three subscales: intrinsic load, F(2, 50) = 13.48, *p* < 0.001, ηp^2^ = 0.350; extraneous load, F(2, 50) = 12.20, *p* < 0.001, ηp^2^ = 0.328; and germane load, F(2, 50) = 13.68, *p* < 0.001, ηp^2^ = 0.354. Bonferroni-corrected post hoc comparisons (Table 19) indicated that intrinsic load was significantly higher in the cognitive overload state than in both the immersion and distraction states, with distraction also exceeding immersion. For extraneous load, the distraction state yielded significantly higher scores than both the cognitive overload and immersion states. For germane load, both the immersion and cognitive overload states showed significantly higher scores than the distraction state. Although the mean value in the immersion state was higher than that in the cognitive overload state, this difference was not statistically significant.

Taken together, these results demonstrate that each experimental state selectively emphasized different components of cognitive load, supporting the effectiveness of the experimental manipulation in inducing distinct cognitive states.

Descriptive statistics for eye-tracking metrics are presented in Table 20. Overall, the cognitive overload state exhibited the highest values across most eye-tracking measures, whereas the immersion state consistently showed the lowest values. As shown in Table 21, the RM-ANOVA revealed significant effects of state on all eye-tracking metrics, including fixation duration, F(2, 50) = 14.37, *p* < 0.001, ηp^2^ = 0.365; fixation count, F(2, 50) = 20.73, *p* < 0.001, ηp^2^ = 0.453; saccade duration, F(2, 50) = 18.29, *p* < 0.001, ηp^2^ = 0.422; and saccade count, F(2, 50) = 19.20, *p* < 0.001, ηp^2^ = 0.434. Bonferroni-corrected post hoc comparisons (Table 22) revealed distinct patterns across states. For fixation-based metrics, both fixation duration and fixation count were significantly higher in the cognitive overload state compared to the immersion state, with distraction showing intermediate values. For saccade-based metrics, the cognitive overload state consistently exhibited higher values than both the distraction and immersion states. In contrast, differences between distraction and immersion were less consistent, with some comparisons not reaching statistical significance. These findings suggest that eye-tracking metrics are sensitive to variations in cognitive states; however, the patterns differ across metrics, indicating that eye movement behavior reflects multiple underlying cognitive processes rather than a single dimension [14,19,36,37].

The relative correlations between the three cognitive load measures and the eye-tracking data were examined using Pearson correlation analysis, and the results are presented in Table 23. The correlation coefficient (r) ranges from –1 to 1, where values closer to 1 indicate a positive correlation, values closer to –1 indicate a negative correlation, and values near 0 indicate no correlation.

The NASA–TLX score showed a weak positive correlation with fixation count (r = 0.27, *p* = 0.01). In addition, the intrinsic cognitive load (IL) score of MCLSVE demonstrated positive correlations with all eye-tracking metrics, including fixation duration (r = 0.26, *p* = 0.05), fixation count (r = 0.31, *p* = 0.01), saccade duration (r = 0.32, *p* = 0.01), and saccade count (r = 0.31, *p* = 0.01). No statistically significant correlations were observed between the other cognitive load measures and eye-tracking variables.

These findings indicate that some cognitive load measures exhibit statistically significant positive relationships with eye-tracking indicators. In particular, the intrinsic cognitive load scale of MCLSVE showed consistent positive correlations across all four eye-tracking metrics, suggesting that intrinsic cognitive load—reflecting task complexity and inherent difficulty—may be more closely associated with gaze behavior. This implies that eye-tracking features are relatively sensitive to capturing aspects of intrinsic cognitive load, particularly those related to cognitive overload during task processing. However, it should be noted that the observed correlation coefficients were generally low, even when statistically significant, and several cognitive load measures did not show significant relationships with eye-tracking data. This suggests that the relationship between cognitive load and eye-tracking metrics may not be adequately captured by simple linear associations.

One possible explanation is that Pearson correlation analysis is limited to detecting linear relationships and may fail to capture more complex, nonlinear patterns present in eye-tracking data. Therefore, the absence of strong correlations should not be interpreted as evidence that eye-tracking data are unrelated to cognitive load. Rather, it suggests that more advanced analytical approaches are required to uncover latent relationships within the data. Based on this limitation, the present study further proposes the use of artificial intelligence-based methods. Deep learning models, such as LSTM, are capable of learning nonlinear and temporal patterns in eye-tracking data, allowing for a more comprehensive and fine-grained analysis of cognitive load compared to traditional statistical methods.

## 6. Development of LSTM Model for Cognitive States Classification

The RM-ANOVA results of eye-tracking data revealed a significant difference among the average eye-tracking data across cognitive states. All eye-tracking metrics exhibited the same average patterns with respect to cognitive states. However, correlation analysis showed that most eye-tracking metrics were not significantly correlated with the cognitive-state scores; moreover, significant correlations were generally weak. Eye-tracking data can significantly vary among individuals and in different situations, giving rise to varying patterns over cognitive states. Therefore, accurately detecting cognitive states using statistical analysis methods (such as mean, standard deviation, and correlation analysis) based on biological signals is difficult. Traditional statistical methods cannot fully capture the characteristics of eye-tracking data, making it challenging to quantify complex cognitive states. AI technologies, which can analyze patterns in eye-tracking data over cognitive states, allow a more effective classification and prediction of cognitive states [11,16,38].

This section presents a deep learning model for classifying learners’ cognitive states based on eye-tracking data. The model was designed to capture temporal patterns in sequential gaze features using a Long Short-Term Memory (LSTM) architecture. The model was implemented in Python 3.8 using the PyTorch framework (2.11.0), along with supporting libraries including pandas, numpy, and scikit-learn. All experiments were conducted on a Windows 10 desktop computer equipped with an Intel Core i7 processor, NVIDIA GeForce RTX 3070 GPU, and 64 GB of RAM. The input dataset consisted of four eye-tracking features: Timestamps, fixation duration, fixation count, saccade duration, and saccade count. Each data point was labeled according to one of three cognitive states: cognitive overload (0), immersion (1), and distraction (2). A total of 7800 data points (2600 per class) were used in the analysis. To construct the training dataset, linear interpolation was applied to align sequences to the length of the longest time series. This study adopted a subject-aware preprocessing strategy to prevent data leakage. Specifically, the dataset was first partitioned based on participants, and sequence data were subsequently generated within each participant’s data. This ensured that temporally overlapping segments from the same individual did not appear simultaneously in both training and test sets. To construct time-series inputs, a sliding window approach was applied within each participant’s data. Each window consisted of 10 consecutive time steps, and the label of each sequence was assigned based on the final time step within the window. This approach preserved the temporal continuity of gaze behavior while avoiding cross-participant contamination. Prior to model training, input features were standardized using the StandardScaler, where the scaler was fitted exclusively on the training data and then applied to the test data. This procedure prevented information leakage from the test set into the training process. To rigorously evaluate model generalizability across individuals, a Leave-One-Subject-Out (LOSO) cross-validation strategy was employed. In each fold, data from one participant were held out as the test set, while the remaining participants’ data were used for training. This approach ensured that the model was evaluated on entirely unseen participants, providing a more realistic assessment of its generalization capability. As shown in Table 24, the LSTM classification model consisted of a single LSTM layer with 50 hidden units, followed by dropout (20%) and batch normalization layers to mitigate overfitting and stabilize training. The final hidden state of the LSTM was passed to a fully connected layer, which output class probabilities through a softmax activation function. The model was trained using the Adam optimizer with a learning rate of 0.001. Sparse categorical cross-entropy was used as the loss function, as class labels were encoded as integers. The model was trained for up to 100 epochs with a batch size of 32. Early stopping was applied based on validation loss to prevent overfitting, and the model parameters corresponding to the lowest validation loss were retained for evaluation.

To evaluate the generalizability of the LSTM model, Leave-One-Subject-Out (LOSO) cross-validation was conducted. In each fold, one participant was used as the test subject, while the remaining participants were used for model training.

Across the 26 LOSO folds, the model achieved a mean accuracy of 75.60% (SD = 13.95%), a mean F1-score of 73.82% (SD = 15.46%), and a mean recall of 75.60% (SD = 13.95%). The mean test loss was 0.9624 (SD = 0.6636). The model also showed strong discriminative performance, with a mean macro-AUC of 86.32 (SD = 12.93%). These results indicate that the LSTM model was able to classify cognitive states from eye-tracking data with moderate generalizability to unseen participants.

**Figure 7:**
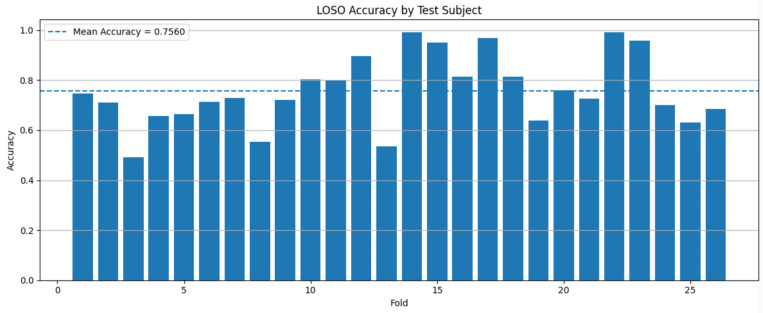
Subject-wise classification accuracy under Leave-One-Subject-Out (LOSO) cross-validation.

Figure 7 presents the classification accuracy for each test subject under the LOSO validation setting. The results show considerable variation across participants. Some test subjects showed very high classification accuracy, exceeding 90%, whereas others showed relatively lower performance, around 40–60%. This suggests that the model’s classification performance was influenced by individual differences in eye-tracking patterns.

As shown in Table 25, class-wise performance was also examined to identify how well the model classified each cognitive state. For cognitive overload, the model achieved a mean precision of 83.50%, recall of 77.12%, and F1-score of 76.68%. For immersion, the mean precision was 78.56%, recall was 74.65%, and F1-score was 72.45%. For distraction, the mean precision was 78.93%, recall was 75.88%, and F1-score was 72.92%. These results suggest that cognitive overload was classified with relatively higher precision and F1-score than the other two states, while immersion showed comparatively lower F1-score.

Overall, the LOSO results indicate that the proposed LSTM model captured meaningful temporal patterns in eye-tracking data for cognitive-state classification. However, the relatively large standard deviations across folds and class-wise metrics suggest that participant-level variability remains an important factor affecting model performance.

**Figure 8:**
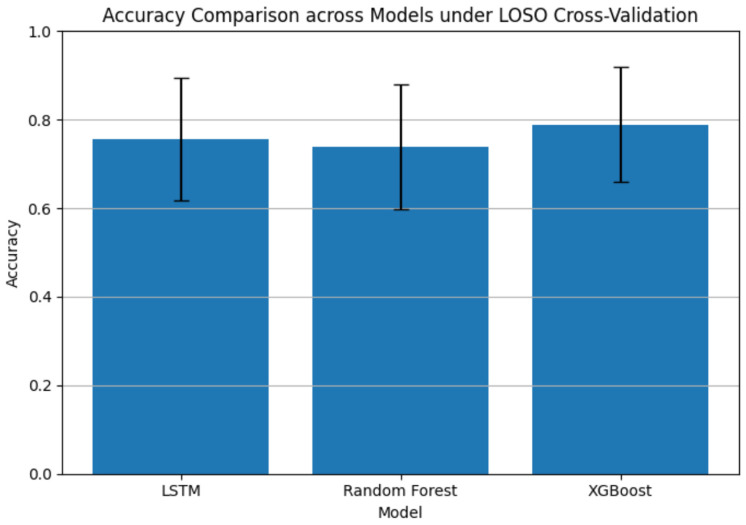
The results of accuracy comparison across models under LOSO cross-validation.

To compare the performance of the proposed LSTM model with baseline approaches, we evaluated random forest and XGBoost under the same LOSO cross-validation framework. The results show that the LSTM model achieved a mean accuracy of 75.60%, outperforming the random forest model (73.80%). The XGBoost model achieved a slightly higher mean accuracy of 78.93%, showing comparable performance to the LSTM model (Figure 8). These findings indicate that while tree-based ensemble methods such as XGBoost can achieve strong classification performance, the proposed LSTM model performs competitively while being specifically designed to capture temporal dependencies in sequential eye-tracking data.

## 7. Discussion and Conclusions

The VR learning environment presents more complex audio–visual stimuli than the traditional 2D-based learning environment, posing a risk of excessive cognitive load that can overstimulate learners and/or distract them from their learning task. To prevent elements that negatively impact the learner’s cognitive states and improve the learning environment, cognitive states must be evaluated during the learning process. Cognitive states can be evaluated subjectively (e.g., utilizing questionnaire responses) or objectively (e.g., using biometric signals). To objectively quantify cognitive states, subjective and objective evaluation methods must be implemented in parallel and correlation between their results must be analyzed. Unlike traditional statistical methods, AI technologies can provide a more accurate quantification of cognitive states.

This study developed a system that evaluated VR learning experiences based on eye-tracking data. The aim was to assess and improve learners’ cognitive states in the VR learning environment. The possible cognitive states in VR—cognitive overload, immersion, and distraction—were categorized and induced in specially designed experimental environments. First, whether each cognitive-state-inducing factor actually elicited the intended cognitive state was determined through a statistical analysis of the learners’ cognitive-state classification scores. The patterns in the eye-tracking data corresponding to each state were also analyzed. To determine the relationships between cognitive states and eye-tracking data, the cognitive-state scores were correlated with the gaze metrics of the participants.

The RM-ANOVA of the cognitive-state assessment scores revealed a significant effect of VR environment states on participants’ cognitive states. The results from subjective measures, including NASA–TLX, FSS, and distractibility, indicated that each state selectively induced its intended cognitive state: cognitive overload was associated with higher perceived cognitive load, immersion with higher flow experience, and distraction with increased distractibility. These findings support the overall validity of the experimental manipulation. In addition, the RM-ANOVA conducted on the eye-tracking data revealed significant effects of state across all four metrics (fixation duration, fixation count, saccade duration, and saccade count). While the cognitive overload state generally exhibited higher values across multiple metrics, the observed patterns varied depending on the specific eye-tracking measure. These results suggest that eye-tracking data are sensitive to differences in cognitive states; however, the relationship is not uniform across metrics. Rather than reflecting a single dimension such as cognitive load, eye movement patterns appear to capture multiple underlying cognitive processes, including task difficulty, attentional allocation, and exploratory behavior.

To further examine the relationship between subjective cognitive-state measures and eye-tracking metrics, correlation analyses were conducted. The NASA–TLX scores of cognitive overload were positively correlated with fixation count, and the intrinsic load subscale of MCLSVE showed positive correlations with all four gaze metrics. However, the other cognitive-state scales were not significantly correlated with eye-tracking metrics. Overall, most eye-tracking indicators showed no significant correlations with cognitive-state evaluation scales, and even the observed correlations were relatively weak in magnitude. These findings suggest that, while certain eye-tracking metrics may reflect aspects of cognitive processing, the relationship between gaze behavior and subjective cognitive-state measures is limited and context-dependent.

To effectively classify and quantify cognitive states using eye-tracking data, an LSTM-based classification model was developed and evaluated using a Leave-One-Subject-Out (LOSO) cross-validation framework. Under this rigorous validation setting, the model achieved a mean accuracy of 75.60% (SD = 13.95%), with a mean F1-score of 73.82% and a mean macro-AUC of 86.32%, demonstrating moderate but reliable generalizability to unseen participants. These results indicate that artificial intelligence techniques, particularly deep learning models capable of capturing temporal dependencies, can effectively quantify cognitive states from eye-tracking data. Although the classification performance showed variability across participants, the model consistently captured meaningful patterns in gaze behavior associated with different cognitive states. This suggests that eye-tracking data contain informative temporal signals that can be leveraged for cognitive-state recognition using AI-based approaches.

The results of the RM-ANOVA fixation duration, count, saccade duration and saccade count tended to be highest in the cognitive overload state, followed by distraction and immersion. These patterns can be interpreted in light of Cognitive Load Theory. Cognitive overload occurs when the demands of a task exceed the learner’s working memory capacity, leading to reduced processing efficiency. Under such states, learners may engage in increased exploratory behavior in an attempt to process and reinterpret information, which can manifest as longer fixation durations and more frequent saccadic movements. In contrast, the immersion state represents a more optimal cognitive state, in which learners are able to focus their attention efficiently on task-relevant information. In this state, visual attention is more stable and selectively directed, resulting in fewer fixations and saccades, as well as more efficient gaze patterns. The distraction state reflects a state in which attention is diverted by irrelevant stimuli. Although this may lead to increased gaze shifts and reduced attentional stability, the overall pattern differs from cognitive overload, as attentional resources are not fully allocated to task processing but are instead distributed across competing stimuli. As a result, eye-tracking metrics in the distraction state tend to fall between those observed in the overload and immersion states.

However, it is important to note that these interpretations should be made with caution. The correlation analysis revealed that the relationships between eye-tracking metrics and subjective cognitive-state measures were generally weak or non-significant. This suggests that eye-tracking data alone may not provide a direct or unambiguous measure of cognitive load. Similar gaze patterns may arise from multiple underlying processes, including exploratory behavior, confusion, task difficulty, or attentional shifts.

Therefore, rather than reflecting a single cognitive construct, eye-tracking metrics should be understood as indicators of complex and context-dependent cognitive processes. This perspective also helps explain the apparent discrepancy between the weak statistical correlations and the classification performance of the LSTM model. While correlation analysis examines linear relationships between individual variables, the LSTM model is capable of capturing complex, nonlinear, and temporal patterns across multiple features. As a result, even when individual eye-tracking metrics show weak associations with subjective measures, their combined patterns over time may still provide sufficient information for distinguishing between cognitive states.

In this study, cognitive states were evaluated from eye-tracking (fixation and saccade) data alone. However, human cognitive states are complex and influenced by various factors that can be derived from a range of physiological signals. For a more comprehensive evaluation of cognitive states, diverse bio-signals such as heart rate and electroencephalogram activity will be incorporated in future work.

In addition, although participants were instructed to minimize head movement during the experiment, head motion was not directly quantified, which may have affected the accuracy of eye-tracking measurements in the VR environment. Future work will address this limitation by incorporating head movement tracking and developing a more robust system that accounts for both eye and head dynamics.

Furthermore, the relatively small sample size (*n* = 26) limits the generalizability of the findings. Given the known variability in eye-tracking behavior across individuals, it is possible that individual differences may have influenced the observed results. Future studies with larger and more diverse samples are needed to improve the robustness and generalizability of the findings.

In addition, the experimental states were designed as composite scenarios that varied across multiple factors, including task difficulty and the presence of audiovisual stimuli. While this approach allowed for the induction of ecologically valid cognitive states in VR environments, it may also introduce potential confounding effects, making it difficult to isolate the influence of individual factors. Therefore, the findings should be interpreted at the level of overall state differences rather than as the effects of specific variables. Future work will aim to disentangle these factors through more controlled experimental designs.

Also, the use of the same subjective measures for both validating cognitive-state induction and examining their relationships with eye-tracking metrics may introduce a potential risk of circular validation. To address this, future research should incorporate independent validation measures or multimodal data sources to more rigorously assess the relationship between cognitive states and physiological signals.

## Figures and Tables

**Table 1:** Eye-tracking data used in the cognitive-state assessment.

Eye-Tracking Data	Implication
Fixation duration, fixation count	Metrics representing the levels of cognitive load, comprehension, and attentional focus.
Saccade duration, saccade count	Metrics representing visual search behavior, information processing, and cognitive activity.

**Table 2:** NASA–TLX evaluation tool.

Category	NASA–TLX Question
Mental Demand	How mentally demanding was the difficulty of the learning content?
Physical Demand	How physically demanding was the difficulty of the learning content?
Temporal Demand	How hurried or rushed was the pace of the learning content?
Effort	How hard did you work to achieve your level while experiencing the learning content?
Frustration	How much of the difficulty of the learning content annoyed, frustrated, or stressed you?
Performance	How successfully were you able to complete tasks while experiencing the learning content?

**Table 3:** Flow State Scale (FSS) used in this study.

FSS Question
I was challenged, and I felt I could meet the challenge.
I did things naturally without thinking too much.
I had a strong sense of what I wanted to do.
I felt I was on track toward my goals.
I was totally focused on what I was doing.
I felt in control of my actions.
It felt like nothing else mattered.
I lost my normal sense of time.
I really enjoyed what I was doing.
I was in the zone.

**Table 4:** Distractibility.

Distractibility Question
How well can you concentrate when distracted by visual elements (background, environment, objects around you, etc.)? *
Do you think you need a quieter environment to learn effectively?
How much did noise inside the virtual reality environment distract you?
Did you have to have quietness around you while you were learning for focusing?
How distracted were you by other students talking loudly while you were learning?
How well were you able to focus on the cell education content during simulation? *
Were you able to focus and not be distracted by ambient noise during simulation? *
How distracted were you when you were with other students inside the virtual reality environment?
How distracting was the audio (sound effects, narration, etc.) inside the virtual reality environment?
How much did the sound of conversations inside the virtual environment distract you?
How distracted were you by students inside the virtual reality environment?
How much did the sound in the virtual reality interfere with your simulation?
Were you able to focus on the cell education without being distracted by other educational content during simulation? *

* = Reflected.

**Table 5:** Multidimensional cognitive load scale for virtual environments (MCLSVE).

Category	MCLSVE Question
IL	The cell education topic covered in the simulation was very complex.
	The simulation covered cell education procedures that I perceived as very complex.
	The simulation covered animal cell concepts and definitions that I perceived as very complex.
EL	Cell instructions and/or explanations used in simulation were very unclear
	Cell instructions and/or explanations used in simulation were, in terms of learning, very ineffective.
	Cell education instructions and/or explanations used in simulation were full of unclear content.
	The interaction technique used in simulation was very unclear.
	The interaction technique used in simulation was, in terms of learning, very ineffective.
	The interaction technique used in simulation made it harder to learn.
	The interaction technique used in simulation was difficult to master.
	The elements (objects, backgrounds, etc.) in the virtual environment made learning very unclear.
	The virtual environment was, in terms of learning, very ineffective.
	The virtual environment was full of content irrelevant to the cell education content.
	It was difficult to find learning information related to cell education in the virtual environment.
GL	The simulation really enhanced my understanding of the cell education topics covered.
	The simulation really enhanced my knowledge and understanding of the structure of animal cells.
	The simulation really enhanced my understanding of the role of each organelle in the animal cell.
	The simulation really enhanced my understanding of the concepts and definitions of animal cells.

**Table 6:** Detection threshold for fixation and saccade.

	Gaze Velocity (v)	Duration
Fixation	v < 30°/s	100 ms ≤ fixation duration ≤ 500 ms
Saccade	v > 60°/s	30 ms ≤ saccade duration ≤ 80 ms

**Table 7:** Cognitive state-inducing elements in the designed experimental environment.

	Cognitive Overload	Distraction	Immersion
Cognitive overload–inducing elements	Complex and difficult	Easy and concise	Easy and concise
Distraction-inducing elements	No audio–visual distractions	Audio–visual distractions unrelated to the cell education content	No audio–visual distractions

**Table 8:** Descriptive statistics results of NASA–TLX.

Cognitive State	NASA–TLX Mean (SD)
Cognitive overload	8.974 (5.393)
Immersion	5.256 (2.29)
Distraction	6.019 (2.554)

**Table 9:** RM-ANOVA results of NASA–TLX.

Measure	F (df1, df2)	*p* (GG-Corrected)	ηp^2^
NASA–TLX	7.95 (2, 50)	0.004 **	0.241

** *p* < 0.01.

**Table 10:** Post hoc comparison results of NASA–TLX.

Comparison	t (df)	*p* (Bonf.)	Cohen’s d	95% CI
Distraction vs. Immersion	1.29	0.621	0.314	[−0.11, 0.79]
Distraction vs. Cognitive overload	−2.60	0.046 *	0.700	[−1.13, −0.28]
Immersion vs. Cognitive overload	−3.30	0.009 **	0.897	[−1.33, −0.55]

* *p* < 0.05, ** *p* < 0.01.

**Table 11:** Descriptive statistics results of FSS.

Cognitive State	FSS Mean (SD)
Cognitive overload	3.804 (0.541)
Immersion	4.269 (0.633)
Distraction	2.992 (1.007)

**Table 12:** RM-ANOVA results of FSS.

Measure	F (df1, df2)	*p* (GG-Corrected)	ηp^2^
FSS	23.42 (2, 50)	<0.001 ***	0.484

*** *p* < 0.001.

**Table 13:** Post hoc comparison results of FSS.

Comparison	t (df)	*p* (Bonf.)	Cohen’s d	95% CI
Distraction vs. Immersion	−5.84	<0.001 ***	1.51	[−2.04, −1.02]
Distraction vs. Cognitive overload	−3.68	0.003 **	1.00	[−1.55, −0.52]
Immersion vs. Cognitive overload	4.52	<0.001 ***	0.79	[0.25, 1.29]

** *p* < 0.01, *** *p* < 0.001.

**Table 14:** Descriptive statistics results of Distractibility.

Cognitive State	Distractibility Mean (SD)
Cognitive overload	2.66 (0.94)
Immersion	2.25 (0.93)
Distraction	5.45 (1.16)

**Table 15:** RM-ANOVA results of Distractibility.

Measure	F (df1, df2)	*p* (GG-Corrected)	ηp^2^
Distractibility	99.74 (2, 50)	0.001 ***	0.800

*** *p* < 0.001.

**Table 16:** Post hoc comparison results of Distractibility.

Comparison	t (df)	*p* (Bonf.)	Cohen’s d	95% CI
Distraction vs. Immersion	10.61	<0.001 ***	3.04	[1.85, 4.15]
Distraction vs. Cognitive overload	10.85	<0.001 ***	2.64	[1.5, 3.62]
Immersion vs. Cognitive overload	−2.60	0.047 *	0.44	[−0.84, −0.12]

* *p* < 0.05, *** *p* < 0.001.

**Table 17:** Descriptive statistics results of MCLSVE.

	Cognitive State	MCLSVE Mean (SD)
IL	Cognitive overload	5.76 (2.46)
	Immersion	3.51 (2.30)
	Distraction	4.45 (2.16)
EL	Cognitive overload	1.82 (1.47)
	Immersion	1.34 (1.67)
	Distraction	2.80 (1.89)
GL	Cognitive overload	6.82 (1.96)
	Immersion	7.66 (2.23)
	Distraction	5.17 (2.65)

**Table 18:** RM-ANOVA results of MCLSVE.

Measure	F (df1, df2)	*p* (GG-Corrected)	ηp^2^
IL	13.48 (2, 50)	<0.001 ***	0.350
EL	12.20 (2, 50)	<0.001 ***	0.328
GL	13.68 (2, 50)	<0.001 ***	0.354

*** *p* < 0.001.

**Table 19:** Post hoc comparison results of MCLSVE.

	Comparison	t (df)	*p* (Bonf.)	Cohen’s d	95% CI
IL	Distraction vs. Immersion	2.52	0.055	0.42	[0.06, 0.76]
	Distraction vs. Cognitive overload	−2.70	0.037 *	0.56	[−0.15, −0.19]
	Immersion vs. Cognitive overload	−5.11	<0.001 ***	0.94	[−1.4, −0.57]
EL	Distraction vs. Immersion	5.18	<0.001 ***	0.82	[0.48, 1.34]
	Distraction vs. Cognitive overload	3.01	0.018 *	0.58	[0.2, 1.02]
	Immersion vs. Cognitive overload	−1.63	0.347	0.31	[−0.7, 0.16]
GL	Distraction vs. Immersion	−4.92	<0.001 ***	1.02	[−1.28, −0.53]
	Distraction vs. Cognitive overload	−3.32	0.008 **	0.71	[−1.13, −0.29]
	Immersion vs. Cognitive overload	1.88	0.215	0.40	[−0.1, 0.85]

* *p* < 0.05, ** *p* < 0.01, *** *p* < 0.001.

**Table 20:** Descriptive statistics results of eye-tracking data.

	Cognitive State	Eye-Tracking Data Mean (SD)
Fixation Duration	Cognitive overload	14.96 (6.31)
	Immersion	6.73 (3.38)
	Distraction	12.98 (7.73)
Fixation Count	Cognitive overload	56.46 (21.39)
	Immersion	24.23 (9.98)
	Distraction	41.42 (21.58)
Saccade Duration	Cognitive overload	3.85 (3.26)
	Immersion	1.02 (0.73)
	Distraction	1.47 (1.09)
Saccade Count	Cognitive overload	60.42 (47.87)
	Immersion	17.96 (12.89)
	Distraction	23.58 (16.43)

**Table 21:** RM-ANOVA results of eye-tracking data.

Measure	F (df1, df2)	*p* (GG-Corrected)	ηp^2^
Fixation Duration	14.37 (2, 50)	<0.001 ***	0.365
Fixation Count	20.73 (2, 50)	<0.001 ***	0.453
Saccade Duration	18.29 (2, 50)	<0.001 ***	0.422
Saccade Count	19.20 (2, 50)	<0.001 ***	0.434

*** *p* < 0.001.

**Table 22:** Post hoc comparison results of eye-tracking data.

	Comparison	t (df)	*p* (Bonf.)	Cohen’s d	95% CI
Fixation Duration	Distraction vs. Immersion	3.95	<0.001 ***	1.05	[0.62, 1.48]
	Distraction vs. Cognitive overload	−1.01	0.957	0.28	[−0.89, 0.3]
	Immersion vs. Cognitive overload	−6.96	<0.001 ***	1.62	[−2.09, −1.08]
Fixation Count	Distraction vs. Immersion	3.69	0.003 **	1.02	[0.5, 1.47]
	Distraction vs. Cognitive overload	−2.51	0.02 *	0.70	[−1.3, −0.12]
	Immersion vs. Cognitive overload	−7.69	<0.001 ***	1.93	[−2.52, −1.35]
Saccade Duration	Distraction vs. Immersion	2.69	0.038 *	0.49	[0.16, 0.85]
	Distraction vs. Cognitive overload	−4.01	0.001 **	0.98	[−1.3, −0.69]
	Immersion vs. Cognitive overload	−4.60	<0.001 ***	1.20	[−1.48, −0.88]
Saccade Count	Distraction vs. Immersion	1.96	0.182	0.38	[−0.01, 0.79]
	Distraction vs. Cognitive overload	−4.21	<0.001 ***	1.03	[−1.37, −0.74]
	Immersion vs. Cognitive overload	−4.70	<0.001 ***	1.21	[−1.54, −0.89]

* *p* < 0.05, ** *p* < 0.01, *** *p* < 0.001.

**Table 23:** Correlations between eye-tracking data and cognitive-state scales.

		Fixation Duration	Fixation Count	Saccade Duration	Saccade Count
NASA–TLX	Pearson Correlation (r)	0.18	0.27 **	0.1	0.11
FSS		−0.18	−0.14	−0.06	0.06
Distractibility		0.17	0.073	−0.14	−0.15
IL		0.26 *	0.31 **	0.32 **	0.31 **
EL		−0.04	−0.06	−0.06	−0.06
GL		−0.01	0.025	0.034	0.05

* *p* < 0.05, ** *p* < 0.01.

**Table 24:** Layer structure of the LSTM model for cognitive-state classification.

Layer	Output Shape	Dropout Rate	Activation Function
Input	(None, 20, 5)	-	-
LSTM	(None, 50)	-	tanh
Dropout	(None, 50)	0.2	-
BatchNormalization	(None, 50)	-	-
Dense	(None, 3)	-	softmax

**Table 25:** Cognitive-state classification scores (precision, recall, and F1-score) of the LSTM model on the test set.

Label	Precision	Recall	F1-Score
Cognitive overload	83.50	77.12	76.68
Immersion	78.56	74.65	72.45
Distraction	78.93	75.88	72.92

## Data Availability

Dataset available upon request from the authors.

## Abbreviations

The following abbreviations are used in this manuscript:

AOI	Area of Interest
AI	Artificial Intelligence
CLT	Cognitive Load Theory
CNN	Convolutional Neural Network
EL	Extraneous cognitive Load
FSS	Flow State Scale
GL	Germane cognitive Load
IL	Intrinsic cognitive Load
LSTM	Long Short-Term Memory
MCLSVE	Multidimensional Cognitive Load Scale for Virtual Environments
NASA–TLX	NASA Task Load Index
I–VT	Velocity–Threshold Identification
VR	Virtual Reality
